# (*E*)-Methyl *N*′-(2-hydroxy­benzyl­idene)­hydrazinecarboxyl­ate at 123 K

**DOI:** 10.1107/S1600536808022332

**Published:** 2008-07-23

**Authors:** Rong Sun, Xiang-Wei Cheng

**Affiliations:** a Zhejiang Police College Experience Center, Zhejiang Police College, Hangzhou 310053, People’s Republic of China

## Abstract

In the title mol­ecule, C_9_H_10_N_2_O_3_, the hydrazinecarboxylic acid mean plane and the benzene ring form a dihedral angle of 11.1 (1)°. In the crystal structure, inter­molecular N—H⋯O hydrogen bonds link the mol­ecules into chains extending along the *b* axis. An intra­molecular O—H⋯N hydrogen bond is also present.

## Related literature

For applications of benzaldehyde­hydrazone derivatives, see: Parashar *et al.* (1988 ▶); Hadjoudis *et al.* (1987 ▶); Borg *et al.* (1999 ▶). For related structures, see: Cheng (2008 ▶).
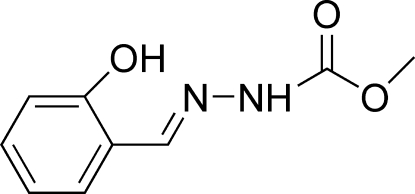


## Experimental

### 

#### Crystal data

C_9_H_10_N_2_O_3_
*M* *_r_* = 194.19
Orthorhombic, 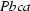

*a* = 9.3998 (17) Å
*b* = 9.0945 (16) Å
*c* = 22.319 (4) Å
*V* = 1908.0 (6) Å^3^
*Z* = 8
Mo *K*α radiation
μ = 0.10 mm^−1^
*T* = 123 (2) K
0.27 × 0.24 × 0.23 mm

#### Data collection

Bruker SMART CCD area-detector diffractometer
Absorption correction: multi-scan (*SADABS*; Bruker, 2002 ▶) *T*_min_ = 0.965, *T*_max_ = 0.968
18306 measured reflections
1679 independent reflections
1427 reflections with *I* > 2σ(*I*)
*R*_int_ = 0.026

#### Refinement

*R*[*F* ^2^ > 2σ(*F* ^2^)] = 0.038
*wR*(*F* ^2^) = 0.120
*S* = 1.05
1679 reflections
128 parameters
H-atom parameters constrained
Δρ_max_ = 0.19 e Å^−3^
Δρ_min_ = −0.19 e Å^−3^

### 

Data collection: *SMART* (Bruker, 2002 ▶); cell refinement: *SAINT* (Bruker, 2002 ▶); data reduction: *SAINT*; program(s) used to solve structure: *SHELXS97* (Sheldrick, 2008 ▶); program(s) used to refine structure: *SHELXL97* (Sheldrick, 2008 ▶); molecular graphics: *SHELXTL* (Sheldrick, 2008 ▶); software used to prepare material for publication: *SHELXTL*.

## Supplementary Material

Crystal structure: contains datablocks I, global. DOI: 10.1107/S1600536808022332/cv2431sup1.cif

Structure factors: contains datablocks I. DOI: 10.1107/S1600536808022332/cv2431Isup2.hkl

Additional supplementary materials:  crystallographic information; 3D view; checkCIF report

## Figures and Tables

**Table 1:** Hydrogen-bond geometry (Å, °)

*D*—H⋯*A*	*D*—H	H⋯*A*	*D*⋯*A*	*D*—H⋯*A*
O1—H1⋯N1	0.84	1.89	2.6234 (16)	145
N2—H2⋯O2^i^	0.88	2.02	2.8881 (16)	169

Symmetry code: (i) 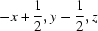.

**Fig. 1.**
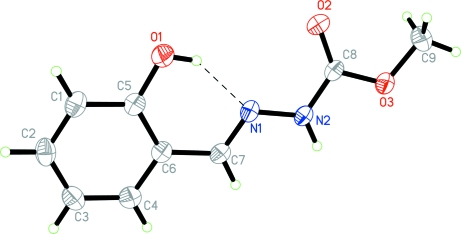
Molecular structure of (I), showing 30% probability displacement ellipsoids and the atomic numbering. Dashed line denotes intramolecular hydrogen bond.

## supplementary crystallographic information

### Comment

Benzaldehydehydrazone derivatives have received considerable attentions for a long time due to their pharmacological activity (Parashar *et al.*, 1988) and their photochromic properties (Hadjoudis *et al.*, 1987). Meanwhile, it's an important intermidiate of 1,3,4-oxadiazoles, which have been reported to be versatile compounds with many useful properties (Borg *et al.*, 1999). As a further investigation of this type of derivatives, the crystal structure of the title compound, C_9_H_10_N_2_O_3_, is described here.

The title molecule (Fig. 1) adopts a *trans* configuration with respect to the C=N bond. Intramolecular O—H···N hydrogen bond (Table 1) influences the molecular conformation. The hydrazine carboxylic acid methyl ester group is slightly twisted away from the attached ring. The dihedral angle between the C1-C6 ring and the C8/C9/N1/N2/O2/O3 plane is 11.1 (1)°. The bond lengths and angles agree with those observed for (*E*)-Methyl *N*'-(4-hydroxybenzylidene) hydrazinecarboxylate (Cheng, 2008).

In the crystal, intermolecular N–H···O (Table 1) hydrogen bonds link the molecules into chains extended along *b* axis.

### Experimental

2-hydroxy benzaldehyde (1.22 g, 0.01 mol) and methyl hydrazinecarboxylate (0.90 g, 0.01 mol) were dissolved in stirred methanol (20 ml) and left for 3 h at room temperature. The resulting solid was filtered off and recrystallized from ethanol to give the title compound in 90% yield. Crystals suitable for X-ray analysis were obtained by slow evaporation of a ethanol solution at room temperature (m.p. 465–468 K).

### Refinement

All H atoms were  positioned geometrically (N—H 0.88 Å, O—H 0.84 Å, C—H 0.95-0.98 Å) and refined in the riding model approximation,, with *U*_iso_(H) = 1.2–1.5*U*_eq_ of the parent atom.

### Crystal data

**Table:**

C_9_H_10_N_2_O_3_	*F*_000_ = 816
*M**_r_* = 194.19	*D*_x_ = 1.352 Mg m^−^^3^
Orthorhombic, *P**b**c**a*	Mo *K*α radiation λ = 0.71073 Å
Hall symbol: -P 2ac 2ab	Cell parameters from 1679 reflections
*a* = 9.3998 (17) Å	θ = 2.0–25.0º
*b* = 9.0945 (16) Å	µ = 0.10 mm^−^^1^
*c* = 22.319 (4) Å	*T* = 123 (2) K
*V* = 1908.0 (6) Å^3^	Block, colourless
*Z* = 8	0.27 × 0.24 × 0.23 mm

### Data collection

**Table:**

Bruker SMART CCD area-detector diffractometer	1679 independent reflections
Radiation source: fine-focus sealed tube	1427 reflections with *I* > 2σ(*I*)
Monochromator: graphite	*R*_int_ = 0.026
*T* = 123(2) K	θ_max_ = 25.0º
φ and ω scans	θ_min_ = 1.8º
Absorption correction: multi-scan(SADABS; Bruker, 2002)	*h* = −10→11
*T*_min_ = 0.965, *T*_max_ = 0.968	*k* = −10→10
18306 measured reflections	*l* = −26→26

### Refinement

**Table:**

Refinement on *F*^2^	Hydrogen site location: inferred from neighbouring sites
Least-squares matrix: full	H-atom parameters constrained
*R*[*F*^2^ > 2σ(*F*^2^)] = 0.038	*w* = 1/[σ^2^(*F*_o_^2^) + (0.0674*P*)^2^ + 0.4536*P*] where *P* = (*F*_o_^2^ + 2*F*_c_^2^)/3
*wR*(*F*^2^) = 0.120	(Δ/σ)_max_ = 0.028
*S* = 1.05	Δρ_max_ = 0.19 e Å^−^^3^
1679 reflections	Δρ_min_ = −0.19 e Å^−^^3^
128 parameters	Extinction correction: SHELXL97 (Sheldrick, 2008), Fc^*^=kFc[1+0.001xFc^2^λ^3^/sin(2θ)]^-1/4^
Primary atom site location: structure-invariant direct methods	Extinction coefficient: 0.018 (2)
Secondary atom site location: difference Fourier map	

### Special details

**Table:**

Geometry. All e.s.d.'s (except the e.s.d. in the dihedral angle between two l.s. planes) are estimated using the full covariance matrix. The cell e.s.d.'s are taken into account individually in the estimation of e.s.d.'s in distances, angles and torsion angles; correlations between e.s.d.'s in cell parameters are only used when they are defined by crystal symmetry. An approximate (isotropic) treatment of cell e.s.d.'s is used for estimating e.s.d.'s involving l.s. planes.
Refinement. Refinement of *F*^2^ against ALL reflections. The weighted *R*-factor *wR* and goodness of fit *S* are based on *F*^2^, conventional *R*-factors *R* are based on *F*, with *F* set to zero for negative *F*^2^. The threshold expression of *F*^2^ > σ(*F*^2^) is used only for calculating *R*-factors(gt) *etc*. and is not relevant to the choice of reflections for refinement. *R*-factors based on *F*^2^ are statistically about twice as large as those based on *F*, and *R*- factors based on ALL data will be even larger.

### Fractional atomic coordinates and isotropic or equivalent isotropic displacement parameters (Å^2^)

**Table:**

	*x*	*y*	*z*	*U*_iso_*/*U*_eq_	
O3	0.37197 (12)	0.41375 (11)	−0.07538 (5)	0.0513 (3)	
O2	0.36215 (12)	0.62287 (11)	−0.02044 (5)	0.0576 (4)	
O1	0.16149 (12)	0.69567 (13)	0.13213 (5)	0.0625 (4)	
H1	0.1906	0.6525	0.1012	0.094*	
N2	0.22056 (13)	0.42471 (13)	−0.00032 (5)	0.0480 (4)	
H2	0.1929	0.3365	−0.0116	0.058*	
N1	0.16212 (12)	0.49157 (13)	0.04907 (5)	0.0452 (3)	
C6	−0.00199 (15)	0.48908 (16)	0.13011 (6)	0.0429 (4)	
C8	0.32185 (15)	0.49856 (15)	−0.03107 (6)	0.0431 (4)	
C5	0.05028 (15)	0.61971 (16)	0.15591 (6)	0.0459 (4)	
C7	0.06038 (16)	0.42561 (15)	0.07632 (6)	0.0461 (4)	
H7	0.0255	0.3347	0.0613	0.055*	
C1	−0.01226 (17)	0.67565 (18)	0.20764 (7)	0.0558 (4)	
H1A	0.0236	0.7634	0.2251	0.067*	
C4	−0.11726 (17)	0.41942 (18)	0.15784 (7)	0.0527 (4)	
H4	−0.1537	0.3311	0.1411	0.063*	
C2	−0.12633 (18)	0.6040 (2)	0.23379 (7)	0.0568 (5)	
H2A	−0.1685	0.6431	0.2690	0.068*	
C3	−0.17925 (17)	0.4760 (2)	0.20895 (7)	0.0573 (4)	
H3	−0.2577	0.4272	0.2269	0.069*	
C9	0.47848 (19)	0.4811 (2)	−0.11312 (7)	0.0617 (5)	
H9A	0.5081	0.4111	−0.1441	0.093*	
H9B	0.5610	0.5087	−0.0887	0.093*	
H9C	0.4386	0.5690	−0.1321	0.093*	

### Atomic displacement parameters (Å^2^)

**Table:**

	*U*^11^	*U*^22^	*U*^33^	*U*^12^	*U*^13^	*U*^23^
O3	0.0596 (7)	0.0459 (6)	0.0484 (6)	0.0025 (5)	0.0093 (5)	−0.0070 (4)
O2	0.0665 (7)	0.0388 (6)	0.0674 (7)	−0.0022 (5)	0.0054 (5)	−0.0070 (5)
O1	0.0605 (7)	0.0593 (7)	0.0678 (7)	−0.0134 (5)	0.0108 (5)	−0.0181 (6)
N2	0.0567 (8)	0.0389 (7)	0.0484 (7)	−0.0020 (5)	0.0070 (6)	−0.0121 (5)
N1	0.0476 (7)	0.0430 (7)	0.0451 (7)	0.0051 (5)	0.0001 (5)	−0.0081 (5)
C6	0.0434 (8)	0.0435 (8)	0.0419 (8)	0.0047 (6)	−0.0069 (6)	0.0001 (6)
C8	0.0478 (8)	0.0366 (7)	0.0448 (8)	0.0075 (6)	−0.0035 (6)	−0.0040 (6)
C5	0.0457 (8)	0.0472 (8)	0.0449 (8)	0.0032 (6)	−0.0052 (6)	−0.0023 (6)
C7	0.0491 (8)	0.0406 (8)	0.0487 (8)	−0.0001 (6)	−0.0032 (6)	−0.0054 (6)
C1	0.0603 (10)	0.0578 (10)	0.0491 (8)	0.0048 (8)	−0.0057 (7)	−0.0124 (7)
C4	0.0526 (9)	0.0508 (9)	0.0548 (9)	−0.0005 (7)	−0.0012 (7)	−0.0002 (7)
C2	0.0586 (9)	0.0722 (11)	0.0395 (8)	0.0159 (8)	−0.0004 (7)	−0.0001 (7)
C3	0.0530 (9)	0.0651 (10)	0.0536 (9)	0.0043 (8)	0.0057 (7)	0.0105 (8)
C9	0.0589 (10)	0.0734 (11)	0.0528 (9)	0.0014 (8)	0.0107 (7)	0.0036 (8)

### Geometric parameters (Å, °)

**Table:**

O3—C8	1.3397 (17)	C5—C1	1.392 (2)
O3—C9	1.4445 (19)	C7—H7	0.9500
O2—C8	1.2158 (18)	C1—C2	1.384 (2)
O1—C5	1.3607 (18)	C1—H1A	0.9500
O1—H1	0.8400	C4—C3	1.380 (2)
N2—C8	1.3522 (19)	C4—H4	0.9500
N2—N1	1.3736 (16)	C2—C3	1.383 (3)
N2—H2	0.8800	C2—H2A	0.9500
N1—C7	1.2823 (19)	C3—H3	0.9500
C6—C4	1.399 (2)	C9—H9A	0.9800
C6—C5	1.409 (2)	C9—H9B	0.9800
C6—C7	1.455 (2)	C9—H9C	0.9800
			
C8—O3—C9	115.49 (12)	C2—C1—C5	120.34 (15)
C5—O1—H1	109.5	C2—C1—H1A	119.8
C8—N2—N1	117.97 (12)	C5—C1—H1A	119.8
C8—N2—H2	121.0	C3—C4—C6	121.58 (15)
N1—N2—H2	121.0	C3—C4—H4	119.2
C7—N1—N2	118.15 (12)	C6—C4—H4	119.2
C4—C6—C5	118.10 (14)	C3—C2—C1	120.42 (15)
C4—C6—C7	119.81 (13)	C3—C2—H2A	119.8
C5—C6—C7	122.09 (14)	C1—C2—H2A	119.8
O2—C8—O3	124.74 (14)	C4—C3—C2	119.56 (16)
O2—C8—N2	125.61 (14)	C4—C3—H3	120.2
O3—C8—N2	109.65 (12)	C2—C3—H3	120.2
O1—C5—C1	117.55 (13)	O3—C9—H9A	109.5
O1—C5—C6	122.45 (13)	O3—C9—H9B	109.5
C1—C5—C6	120.00 (14)	H9A—C9—H9B	109.5
N1—C7—C6	120.40 (13)	O3—C9—H9C	109.5
N1—C7—H7	119.8	H9A—C9—H9C	109.5
C6—C7—H7	119.8	H9B—C9—H9C	109.5

### Hydrogen-bond geometry (Å, °)

**Table:**

*D*—H···*A*	*D*—H	H···*A*	*D*···*A*	*D*—H···*A*
O1—H1···N1	0.84	1.89	2.6234 (16)	145
N2—H2···O2^i^	0.88	2.02	2.8881 (16)	169

Symmetry codes: (i) −*x*+1/2, *y*−1/2, *z*.
